# Identification of Quantitative Trait Loci Conditioning the Main Biomass Yield Components and Resistance to *Melampsora* spp. in *Salix viminalis* × *Salix schwerinii* Hybrids

**DOI:** 10.3390/ijms18030677

**Published:** 2017-03-22

**Authors:** Paweł Sulima, Jerzy A. Przyborowski, Anna Kuszewska, Dariusz Załuski, Małgorzata Jędryczka, Witold Irzykowski

**Affiliations:** 1 Department of Plant Breeding and Seed Production, University of Warmia and Mazury in Olsztyn, Plac Łódzki 3, 10-724 Olsztyn, Poland; jerzy.przyborowski@uwm.edu.pl (J.A.P.); anna.kuszewska@uwm.edu.pl (A.K.); dariusz.zaluski@uwm.edu.pl (D.Z.); 2 Institute of Plant Genetics, Polish Academy of Sciences, Strzeszyńska 34, 60-479 Poznań, Poland; mjed@igr.poznan.pl (M.J.); wirz@igr.poznan.pl (W.I.)

**Keywords:** *Salix*, QTL, biomass willow, leaf rust resistance

## Abstract

The biomass of *Salix viminalis* is the most highly valued source of green energy, followed by *S. schwerinii*, *S. dasyclados* and other species. Significant variability in productivity and leaf rust resistance are noted both within and among willow species, which creates new opportunities for improving willow yield parameters through selection of desirable recombinants supported with molecular markers. The aim of this study was to identify quantitative trait loci (QTLs) linked with biomass yield-related traits and the resistance/susceptibility of *Salix* mapping population to leaf rust. The experimental material comprised a mapping population developed based on *S. viminalis* × *S. schwerinii* hybrids. Phenotyping was performed on plants grown in a field experiment that had a balanced incomplete block design with 10 replications. Based on a genetic map, 11 QTLs were identified for plant height, 9 for shoot diameter, 3 for number of shoots and 11 for resistance/susceptibility to leaf rust. The QTLs identified in our study explained 3%–16% of variability in the analyzed traits. Our findings make significant contributions to the development of willow breeding programs and research into shrubby willow crops grown for energy.

## 1. Introduction

Species of the genus *Salix* spp. have unique properties which make them suitable for different practical applications [1,2,3]. Shrubby willows have numerous practical benefits, including renewable energy generation, due to their high yield potential and rapid regrowth after cutting. The above properties increase the popularity of willow biomass plantations in many countries in Europe and North America [4]. *Salix viminalis* is the most highly valued source of green energy, followed by *S. dasyclados*, *S. schwerinii*, *S. eriocephala*, *S. burjatica*, *S. pentandra*, *S. triandra* and selected interspecies hybrids [5]. Considerable variability in productivity observed in the genus *Salix* [6] creates new opportunities for improving yield parameters through selection and breeding [7]. These opportunities have been confirmed by molecular analyses, which revealed high levels of genetic diversity between willow species [8,9,10], contributing to the successful choice of parental components for crossing and the probability of achieving valuable hybrids. Varieties intended for energy generation should be characterized by high biomass yield, resistance to common diseases and pests, high tolerance for changing environmental conditions and desirable quality of wood, which increases its energy value [9,11].

Molecular marker types play an important role in contemporary creative breeding, including of high-yielding varieties of willow. Molecular markers are used at many breeding steps, which increases the effectiveness of breeding programs and considerably shortens breeding cycles [12]. Genetic maps and the identification of quantitative trait loci (QTLs) responsible for the traits that determine willow’s suitability for energy generation are highly useful tools in breeding. The progress made in genetics, genomic research and phenotyping significantly expanded our knowledge about the formation of yield-related traits, including biomass, and contributed to precision breeding by marker-assisted selection (MAS) [7]. Linkage mapping and physical mapping provide basic information about genes encoding important traits, and they set objective criteria for selecting parental material. A few genetic maps supporting the identification of QTLs linked with various traits have been developed for the genus *Salix* [8,13,14,15,16,17]. In willows, most of the identified QTLs were linked with biomass yield and yield-associated traits such as plant height, shoot diameter or side branching [17,18,19,20,21]. Molecular markers linked to leaf rust resistance [22], sex [23], phenological traits [14,19], elemental composition of biomass [24], tolerance for environmental factors [18,25], and resistance to diseases and pests [22,26,27] were also identified. Quantitative trait loci associated with plant resistance also play an important role in willows, in particular in varieties intended for large-area multiannual plantations, which become increasingly susceptible to diseases with time. One of the key pathogens in willows are fungi of the genus *Melampsora* which cause leaf rust. According to Parker et al. [28], this disease can reduce willow yield by up to 40%. Attempts are being made to find natural sources of resistance against this pathogen [29] and to identify QTLs linked with resistance to leaf rust in willows [22]. Despite the significant progress that has been made in recent years, the existing knowledge about the genetic background of yield-related traits, willow yields and resistance to leaf rust in willows needs to be expanded. Further efforts are required to generate genetic maps based on various marker systems and mapping populations derived from interspecific crosses. The relevant research aims to accurately identify all QTLs and important performance traits. Therefore, the main objective of this study was to identify quantitative trait loci linked with the main yield-related traits of *Salix* mapping population and their resistance/susceptibility to *Melampsora larici-epitea*.

## 2. Results

### 2.1. Yield-Associated Traits

**Figure 1:**
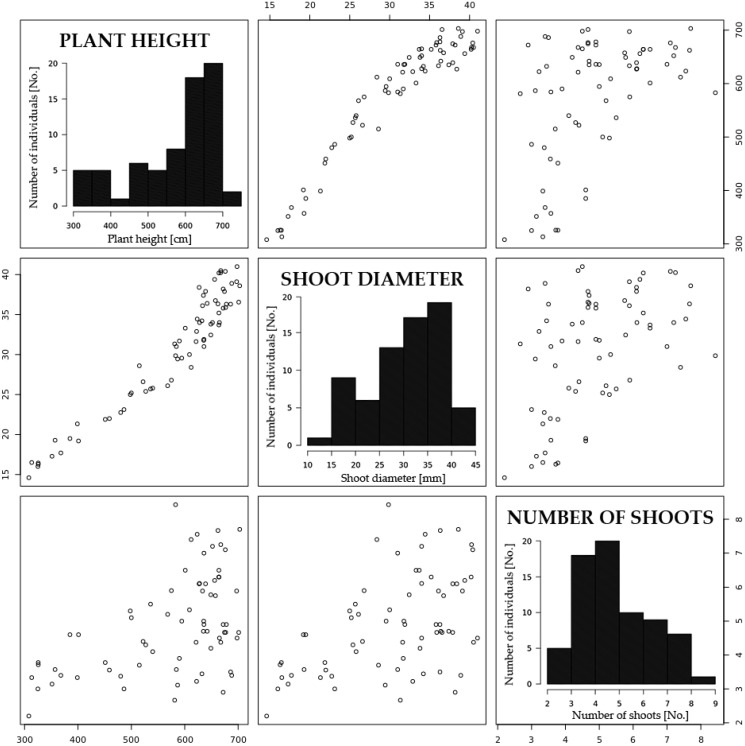
Distributions and scatter plots of phenotypic means for basic yield-associated traits of P5 mapping population. In the case of graphs showing the relationships between the traits, the coordinate axes located outdoors of charts describe the values of individual traits.

Throughout the years of the research, the analysis of variance (ANOVA) has indicated significant differences (*p* < 0.01) between the individuals comprising population P5 for all the analyzed traits: plant height, shoot diameter and the number of shoots per plant. The variability in basic yield-associated traits increased in successive years of the field experiment. Based on the calculated coefficients of variability, the highest variability was observed in three-year-old plants (Table 1). Despite the above, the analyzed traits after checking by Shapiro-Wilk W-test were close to normally distributed with negative skew (plant height and shoot diameter) and positive skew (number of shoots) (Figure 1). A highly significant positive correlation was observed between plant height and shoot diameter (*r* = 0.96). The correlations between the number of shoots and plant height and between the number of shoots and shoot diameter were not statistically significant (*p* ≤ 0.05).

### 2.2. Resistance/Susceptibility to Willow Rust

The tested isolates of *M. larici-epitea* (Mle1–Mle4) were differed in their pathogenicity toward plants of the mapping population. In cases of severe infection with isolate MIe4, 40.5% of leaf disc area was covered with uredinia; in leaves inoculated with isolate MIe3, infected leaf area was 10.71%, whereas the remaining two isolates caused intermediate symptoms of infection (Table 2). There were no statistical differences (*p* ≤ 0.05) between the biological replicates of the same isolate, i.e. the same test repeated twice resulted in the same plant reaction to the tested isolates.

**Figure 2:**
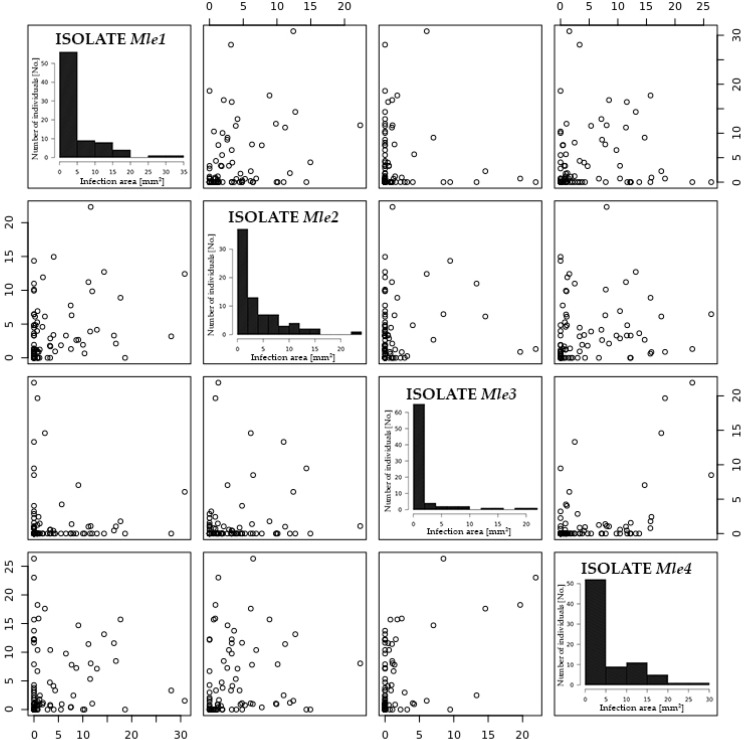
Distributions and scatter plots of phenotypic means for infected area on the leaf disc tested hybrids of P5 population.

Leaf disc area colonized by the four *Melampsora* isolates was not characterized by normal distribution of data (Figure 2), both with and without transformation. The distribution of this trait was significantly skewed to the right. Despite the above, non-transformed data were subjected to QTL mapping, and a similar approach had been adopted by Samils et al. [22]. The cited authors and Van Ooijen [30] found that QTL mapping by maximum-likelihood estimation is quite robust against deviations from normal distribution.

### 2.3. Linkage Analysis and Mapping

**Figure 3:**
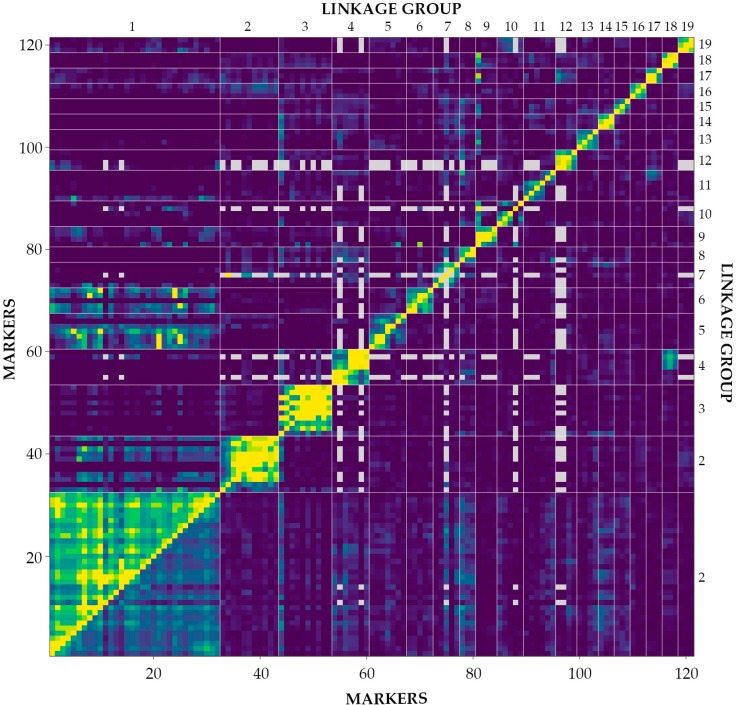
Plot of estimated recombination fractions (**upper left** triangle) and LOD scores (**lower right** triangle) for all pairs of markers in the linkage map of the population P5.

**Figure 4:**
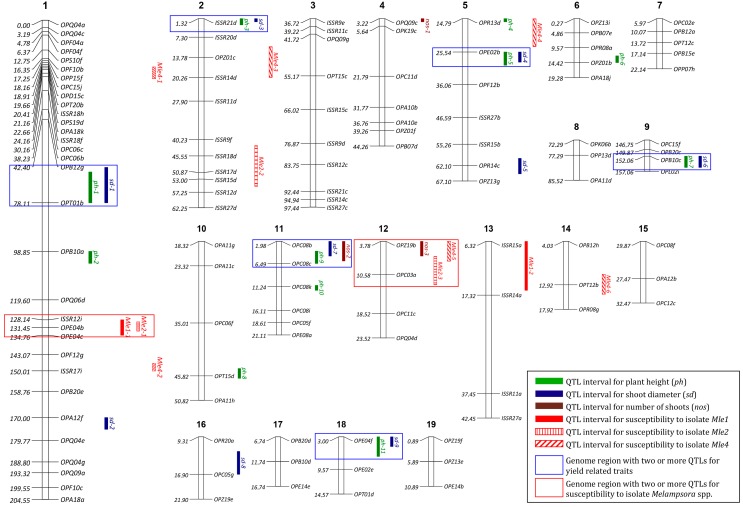
Genetic map of the population P5 (*Salix viminalis* × *Salix schwerinii*) and locations of detected quantitative trait loci (QTLs). Numbers on the left of each linkage group (1–19) show absolute marker position in Haldane map units. Markers names are to the right.

A total of 419 primers (300 RAPD—Randomly Amplified Polymorphic DNA and 119 ISSR—Inter Simple Sequence Repeats) were tested, and 85 RAPD and 9 ISSR primers, respectively, were selected for further analyses. A total of 724 markers, including 539 (74.4%) polymorphic markers, were identified (Table 3). In the group of polymorphic markers validated with missing parent alleles, duplicate markers and segregation patterns (a total of 387 markers) were used in linkage analysis. Only the markers from linkage groups with minimum three markers (excluding doublets and unlinked markers) were used to generate a genetic map. Markers in linkage groups were ordered, rippled, and re-ordered according to pairwise recombination fractions, LOD scores (Logarithm of Odds) (Figure 3) and linkage group length. The genetic map contained 121 markers in 19 linkage groups corresponding to the number of chromosomes characteristic of the analyzed *Salix* mapping population (Figure 4). The size of those groups ranged from 10.0 to 204.5 cM. The largest linkage group contained 32 markers. The total length of the generated map was 656.4 cM, and the average distance between markers was 6.3 cM, with the maximal spacing 35.7 cM (Table 3, Figure 4).

### 2.4. Identification of QTLs

A total of eleven QTLs in eight linkage groups were identified for plant height (Table 4, Figure 4). LOD values for different QTLs ranged from 4.48 to 13.20 with 4.13 LOD thresholds (1000 permutations, α = 0.05) and explained 2.97% to 9.72% of variability in this trait. Nine QTLs in seven linkage groups were identified for shoot diameter, with LOD values of 3.27–6.07, and they explained 4.10%–7.90% of variability. Interestingly, a high number (six) of QTLs linked with shoot diameter were localized on the map in the proximity of or in the same locus as the QTLs linked with plant height. Three QTLs linked with the number of shoots per stump were identified with LOD values of 3.38–5.08 and 10.37%–16.16% of explained variability in this trait. One of the QTLs linked with the number of shoots per plant (*nos-2*) was localized in the proximity of *sd-7* and *ph-9*.

In three out of the four analyzed isolates, the results of disc infection tests were used to identify 11 QTLs in six linkage groups associated with the severity of leaf rust (statistically significant QTLs were identified for all isolates, except Mle3). Four of those QTLs were found in group 1 (Figure 4). Fungal isolate Mle4 deserves special attention in the process of QTL identification because it was characterized by the highest pathogenicity (Table 2), which was also correlated with the highest number of QTLs associated with the severity of leaf rust, which were identified with the use of that isolate (Table 4). The LOD values of QTLs identified for isolate Mle4 ranged from 6.50 to 19.50, and they were highest relative to the remaining isolates. In two cases, QTL pairs localized in close vicinity (Mle1–1 and Mle2–1, Mle2–3 and Mle4–5) were observed. All QTLs associated with the severity of leaf rust explained 4.46% to 15.47% of phenotypic variability. 

## 3. Discussion

In this study, the simplest marker systems, RAPD and ISSR, were used on account of their ease of use, rapidity, low cost, low labor and low hardware requirements and applicability when DNA sequences are not known. RAPD and ISSR systems are universal tools that are highly useful at various stages of breeding new plant varieties. In the existing studies, willow loci were identified mainly in analyses of biomass yield [17,18,19,20,21]. Quantitative trait loci linked with the resistance/susceptibility of *Salix* species to leaf rust were rarely identified, including by Rönnberg-Wästljung et al. [31], Hanley et al. [26] and Samils et al. [22]. The cited authors developed QTL maps with the application of AFLP, RFLP, SNP and SSR markers. In the present experiment, a higher number of QTLs linked with plant height, shoot diameter and number of shoots per plant (11, 9 and 3 respectively) was identified than in other studies that relied on different types of markers [17,19]. The identified QTLs explained a lower percentage of variability in the analyzed traits (2.97%–16.16%) than those identified by other authors, which explained 14%–22% [17], 10%–20% [21] and 8%–42% of variability [18]. The results of the cited studies suggest that the analyzed traits are controlled by many loci of limited individual impact, as noted by Hallingbäck et al. [19]. It should be stressed that our experiment had a balanced incomplete block design with 10 replications. Small incomplete blocks minimized the effect of soil variability, and complete balancing minimized competition effects because every experimental treatment neighbored the same treatment only once. An experimental design with a high number of replications made it easier to control the variation of numerous factors that significantly affected the analyzed traits. The chosen experimental design could have significantly influenced the percentage of phenotypic variability explained by each QTL and the number of identified QTLs. Only three statistically significant QTLs identified for the number of shoots per plant could indicate that this trait is weakly inherited and conditioned by a smaller number of loci, which, despite a relatively high percentage of explained variability in that trait, suggests that its value is more likely to be influenced by environmental factors than other traits. It should also be noted that the number of shoots in perennial plants such as willows is more likely to fluctuate across years than other examined traits due to variations in winter weather. To date, only four QTLs linked with the number of shoots per plant have been described, including three SNPs [19] and one AFLP/RFLP [17].

In this study, special attention was also paid to several genomic regions where more than one QTL controlling yield-related traits was identified. The OPC08b–OPC08c (1.98–6.49 cM) interval in linkage group 11, which included three QTLs determining three yield-related traits (*ph-9*, *sd-7* and *nos-2*), seemed to be most pertinent to biomass production. *nos-2* also explained the highest percentage of variability in the number of shoots per plant (16.16%). In addition, five genomic regions (in the vicinity of markers OPT-01b, ISSR-21d, OPE-02b, OPB-10c and OPE-04f) revealed the proximity of QTLs linked with plant height and shoot diameter.

The QTLs linked with resistance/susceptibility to *Melampsora* spp. were identified based on a new parameter, namely infected area on the leaf disc (see Section 2.4). This parameter was used to identify QTLs because it is the most robust indicator of infection severity, which was highly significantly correlated with the diameter of uredinia in our study. The number of identified QTLs linked with resistance to leaf rust (11) was lower than that reported in other studies. The percentage of phenotypic variability was also lower in this study (4.5%–15.5%). Rönnberg-Wästljung et al. [31] analyzed two mapping populations (BC and F2) and identified 16 QTL regions linked with the number of uredinia, their diameters and incubation periods, and phenotypic variability associated with QTL was explained in 8%–26%. Hanley et al. [26] studied mapping population K8 to analyze leaf rust infection under field conditions, and the number and diameter of uredinia. The cited authors used the interval method to identify 22 QTL regions which explained phenotypic variability in up to 56.4%. Samils et al. [22] analyzed two mapping populations (BC and F1) and identified 28 QTLs linked with parameters describing resistance to leaf rust, such as field infections, number and diameter of uredinia, incubation period and flecking with three rust strains. The percentage of explained variability ranged from 5.5% to 56.2%. This study identified two genomic regions, each containing two QTLs linked with resistance/susceptibility to leaf rust, which increases their significance in the search for individuals resistant to *Melampsora* spp. The interval between markers OPZ-19b do OPC-03a and the second interval in the vicinity of marker OPE-04b together explained 34.68% of phenotypic variability in this trait (13.32% and 21.36%, respectively).

In this study, the results of QTL analyses were significantly influenced by the type of inoculation isolate, and similar observations were made by Samils et al. [22] and Rönnberg-Wästljung et al. [31]. Fungal isolate Mle4 supported the identification of six QTLs with the mean LOD of 13.13. Significantly lower LOD values were determined for QTLs identified for the remaining isolates. A strong positive correlation was also observed between an isolate’s pathogenicity and the number of identified QTLs linked with resistance/susceptibility to leaf rust. No QTLs were identified in isolate Mle3 which was characterized by the lowest pathogenicity in willow plants. This indicates that isolates with the highest pathogenicity should be selected for the identification of QTLs associated with resistance/susceptibility to leaf rust.

The aim of plantations growing willows for bioenergetics is to improve biomass yield. Breeding programs focus on the identification of QTLs conditioning the major yield-related traits, mostly plant height, shoot diameter, number of shoots per plant, and resistance to pathogens. The results of this study make a valuable contribution to research into willows grown for energy purposes. The identification of numerous QTLs, which explain a high percentage of phenotypic variability of important yield-associated traits, provides new insights into the field and supplements the existing knowledge base. Our results support the introduction of marker-assisted selection into the existing and future willow breeding programs. Despite numerous achievements in this field, further research is needed to expand our knowledge of the genome of willows grown for biofuel.

## 4. Materials and Methods

### 4.1. Plant Material

The experimental material comprised 79 willows of mapping population P5 which was developed in the Department of Plant Breeding and Seed Production of the University of Warmia and Mazury in Olsztyn (Poland) by backcrossing a female form of cv. Tordis ((*S. schwerinii* × *S. viminalis*) × *S. viminalis*) with a randomly selected male form Z1/13, an F1 hybrid of Tordis × Torhild ((*S. schwerinii* × *S. viminalis*) × *S. viminalis*). A field experiment was set up in Baldy near Olsztyn (53°36′01″ N 20°36′14″ E) in 2010. The experiment had a balanced incomplete block design with 10 replications in accordance with plan No. 11.46 proposed by Cochran and Cox [32]. Willows were planted with 70 cm × 70 cm spacing to eliminate the edge effect. The experimental plants were surrounded by an additional row of willows from the mapping population.

### 4.2. Evaluation of Yield-Associated Traits

Biometric parameters, including the height and diameter of the main stem (plant height (*ph*) and steam diameter (*sd*)) and the number of shoots per plant (*nos*), were measured after each growing season. Quantitative trait loci were identified based on measurements of 3-year-old plants growing on a 4-year-old stump. The empirical distribution of the analyzed traits was checked for normality in the Shapiro-Wilk *W*-test [33]. The results were processed by one-way ANOVA. The significance of differences between means was analyzed by Tukey’s HSD test, a multiple comparisons procedure, at *p* ≤ 0.05. All calculations were performed in Statistica v. 12.5 (Statistica, Tulsa, OK, USA) [34]. 

### 4.3. Fungal Material

*Melampsora larici-epitea* isolates for leaf disk tests under laboratory conditions were obtained from samples collected in the experimental field located in Baldy. The inoculum (spore suspension) was obtained from genetically identical spores. The spores were collected from willow leaves showing the symptoms of rust and propagated by spreading the spores originating from a single pustule on the leaves of willow hybrids susceptible to leaf rust (*S. aurita* L. × *viminalis* L. × *caprea* L.). The inoculated leaves were placed with the upside down on Petri plates lined with moistened filter paper. The spore suspension was used to inoculate plants in a controlled environment. The plates were placed in WB 1500 KFL controlled environment chambers (Mytron Bio- und Solartechnik GmbH, Heiligenstadt, Germany) at a temperature of 16 °C, 80% relative air humidity and a 12 h/12 h photoperiod. The spores were shaken off leaves onto aluminum foil, and they were dried for 3–4 days at room temperature. The described propagation cycle of fungal isolates was repeated until a sufficient quantity of spores was obtained for inoculation tests (15–20 mg).

Spores were identified as belonging to the species of *M. larici-epitea* based on morphological and genetic analyses. The morphological traits of spores (size, shape and location of spikes on the surface) were described under a scanning electron microscope, and genetic analysis involved sequencing of ITS1-5 and 8S-ITS2 (internal transcribed spacer) regions. The DNA barcodes, specifically those defined on ITS, provided a highly accurate means of identifying and resolving *Melampsora* taxa of aspen and white poplar [35]. The same barcode was used in this study, in addition to morphological characterization of the spores.

### 4.4. Evaluation of Plant Resistance

The resistance of the tested plants (P5 population) was evaluated on leaf discs with a diameter of 20 mm. Leaf discs were placed on square Petri plates with 25 compartments (Sterilin Limited, ThermoFisher Scientific, Cambridge, UK) lined with filter paper segments (Whatman 3MM, GE Healthcare, Maidstone, UK). Leaf discs representing different genotypes were inoculated with four *M. larici-epitea* isolates (Mle1–Mle4), in five technical replications and two biological replications each. The suspension of freshly propagated spores at a concentration of 1–2 × 10^5^/mL water, containing 0.004% of the Tween 20 detergent, was placed on Petri plates (10 cm × 10 cm) in the amount of 1 mL per plate with the use of an air brush with a 0.35 mm nozzle (Air Brush Kit, EW-6000B, 0.2 mm, Jadar Model, MAR, Warsaw, Poland). The viability and germination capacity of fungal spores were checked under the light microscope after 24 and 48 h of incubation in 0.5% aqueous agar solution. The severity of fungal infection was evaluated in the ImageJ program (imagej.net, National Institutes of Health, Bethesda, MD, USA) for image processing and analysis. The number and surface area of uredinia were determined, and the results were used to calculate leaf area colonized by fungi. The evaluation was done 13 days post inoculation (13 dpi). The analysis relied on an infected area on the leaf disc, which was calculated by multiplying the number of uredinia by their surface area. If the normal distribution hypothesis for this trait was rejected, data were transformed by the method proposed by Bliss [36]

### 4.5. RAPD and ISSR Markers

DNA was isolated from young willow leaves with the modified method described by Sulima et al. [37]. The content and quality of DNA were evaluated with the use of the Nano Drop 2000 UV-vis spectrophotometer (Thermo Fisher Scientific, Waltham, MA, USA). DNA was prepared with identical concentrations of the matrix (10 ng per 1 µL).

PCR-RAPD [38] and PCR-ISSR [39] reactions were carried out in the Universal Gradient peqSTAR 96 thermocycler (Peqlab Biotechnologie GmbH, Erlangen, Germany). The reaction mixture with a volume of 25 µL had the following composition: 2.5 µL of the PCR buffer (10× DreamTaq™ Green Buffer, Fermentas Thermo Scientific, Waltham, MA, USA), 0.6 nM of dNTP (Sigma Aldrich Srl, Milan, Italy), 0.4 mM of the primer, 0.5 U of DreamTaq DNA polymerase (Fermentas Thermo Scientific, Waltham, MA, USA), 10 ng of DNA and sterile distilled water. In both methods, the reaction was carried out in 35 cycles with initial denaturation at 94 °C for 1 min and final elongation at 72 °C for 10 min. The PCR-RAPD reaction had the following thermal profile: I—denaturation at 94 °C for 30 s; II—primer annealing at 40 °C for 2 min; III—elongation at 72 °C for 2 min. The sequence of ISSR primers was selected based on the work of McGregor et al. [40]. Before ISSR primers were selected, the optimal annealing temperature was determined for each primer, and amplification trials were carried out to selected primers that generate clear and repeatable products. Nine primers with annealing temperatures of: ISSR 12 and 17, 55.5 °C; ISSR 20, 58.0 °C; ISSR 27, 59.0 °C; and ISSR 15, 17, 21 and 28, 60.0 °C were selected for mapping.

Amplification products were separated in 1.5% agarose gel with ethidium bromide (Sigma Aldrich Chemie GmbH, Steinheim, Germany). They were visualized in UV light in the DIGIDOC imaging system (Biogenet, Warsaw, Poland). Each genotype was analyzed twice. The size of amplification products was determined in the TotalLab100 program (Nonlinear Dynamics, Newcastle, UK). The applied standards were the GeneRuler™ 100 bp Plus DNA Ladder (100–3000 bp) and the GeneRuler™ 100 bp DNA Ladder (Fermentas Thermo Scientific, Waltham, MA, USA).

### 4.6. Linkage Analysis, Genetic Mapping and QTL Mapping

Linkage analysis was performed in the R/QTL [41], using the procedure described in “Genetic map construction with R/QTL” by Broman and Sen [42]. Data were prepared for mapping by excluding missing parent alleles, duplicate markers, markers with call rates of less than 0.75, and markers not exhibiting Mendelian segregation. The missing data for the analyzed individuals did not exceed 10%, and all data were included in the analysis which covered a total of 463 markers and 79 individuals. Markers were assigned to linkage groups for analysis as a phase-known four-way cross [43] in R/QTL software (University of Wisconsin-Madison, Madison, WI, USA) using the *formLinkageGroups* function (max.rf. = 0.35 min, LOD = 5). Markers were ordered, rippled, and re-ordered according to pairwise recombination fractions, LOD scores and linkage group length.

The QTLs responsible for biomass yield-related traits and leaf area infected by fungi were identified by maximum-likelihood interval mapping with the use of the EM algorithm [44] in R/QTL software using the procedure described by Broman and Sen [42]. QTL genotype probability was calculated using the *calc.genoprob* function with a step size of 1 cM. Simple interval mapping analyses of each separate trait were first performed to detect potential QTL positions using the *scanone* function. Genome-wide LOD significance thresholds were calculated based on 1000 permutations at 0.05 α-value level [45]. For each trait, two-dimensional genome scans were performed for the two-QTL model (*scantwo* function) to identify successive QTLs, and their location on the genetic map was optimized (*makeQTL*, *fitQTL*, *refineQTL* and *addQTL* functions). Interval mapping was performed by calculating the 95% Bayesian credible interval [46] with the *bayesint* function in R/QTL software.

The percentage variability in a phenotypic trait explained by a given single QTL was assessed using the *fitqtl* function. The identified QTLs were mapped in the MapChart 2.3 application (Kyazma BV, Wageningen, The Netherlands) [47].

## Figures and Tables

**Table 1:** Biometric features of hybrids crop in three subsequent growing seasons (2011–2013).

Trait	Plants One-Year	Plants Two-Year	Plants Three-Year
Plant height (cm)	346.3 ± 34.2 (9.9%) ^1^	436.6 ± 57.1 (13.1%)	576.9 ± 114.6 (19.9%)
Shoot diameter (mm)	19.1 ± 2.8 (14.7%)	21.6 ± 3.5 (16.2%)	30.7 ± 7.5 (24.4%)
Number of shoots (No.)	8.6 ± 2.4 (27.9%)	6.8 ± 1.7 (25.0%)	4.9 ± 1.5 (30.6%)

^1^ Mean ± standard deviation (coefficient of variability).

**Table 2:** Main characteristics of susceptibility to the isolates of *Melampsora larici-epitea* used in the study.

Isolate Name	Leaf Area Covered with Uredinia (%)	Mean Area of a Pustule (mm^2^)
Mle1	25.41 ^c^ ^1^	1.773 ^c^
Mle2	32.14 ^b^	1.405 ^b^
Mle3	10.71 ^d^	0.617 ^d^
Mle4	40.49 ^a^	2.355 ^a^

^1^ Letters show statistical differences between the isolates in respect to variable infection of the tested hybrids of P5 population, based on Tuckey’s test (*p* < 0.05).

**Table 3:** Summary of results from the linkage analysis.

Parameter	Mapping Population
	P5
Total markers	724
Average markers per primer	5.7
RAPD ^1^ markers used for linkage analysis	93
ISSR ^2^ markers used for linkage analysis	28
Total markers used for linkage analysis	121
Unlinked or doublets markers	340
Number of groups in the framework	19
Smallest group (cM) of the framework	10.0
Largest group (cM) of the framework	204.5
Average length (cM) of group	34.5
Total length (cM) of the framework	656.4
Average distance between two framework markers (cM) ± SD	6.3 ± 1.9

^1^ RAPD—Randomly Amplified Polymorphic DNA; ^2^ ISSR—Inter Simple Sequence Repeats.

**Table 4:** QTLs associated with studied traits detected by interval mapping in population P5.

Trait	LOD Threshold	QTL Name	Nearest Marker	LG ^1^	Position (cM)	LOD	%PVE ^2^
Plant height	4.13	*ph-1*	OPT01b	1	67.00	4.48	2.97
		*ph-2*	OPB10a	1	103.00	5.73	3.85
		*ph-3*	ISSR21d	2	2.32	6.77	4.61
		*ph-4*	OPR13d	5	14.79	8.27	5.73
		*ph-5*	OPE02b	5	28.79	6.99	4.78
		*ph-6*	OPZ01b	6	13.27	10.51	7.49
		*ph-7*	OPB10c	9	154.75	13.20	9.72
		*ph-8*	OPT15d	10	45.82	11.27	8.10
		*ph-9*	OPC08c	11	4.98	7.19	4.92
		*ph-10*	OPC08k	11	11.24	5.59	3.75
		*ph-11*	OPE04f	18	6.01	6.62	4.50
Shoot diameter	3.26	*sd-1*	OPT01b	1	64.00	3.86	4.10
		*sd-2*	OPA12f	1	174.00	3.50	4.88
		*sd-3*	ISSR21d	2	1.32	5.03	4.40
		*sd-4*	OPE02b	5	27.79	6.07	6.46
		*sd-5*	OPR14c	5	60.79	3.71	7.90
		*sd-6*	OPB10c	9	154.75	3.95	4.52
		*sd-7*	OPC08b	11	3.98	3.27	4.68
		*sd-8*	OPC05g	16	13.31	3.59	4.65
		*sd-9*	OPE04f	18	4.00	3.69	4.99
Number of shoots	3.19	*nos-1*	OPQ09c	4	4.22	3.45	10.60
		*nos-2*	OPC08b	11	1.98	5.08	16.16
		*nos-3*	OPZ19b	12	3.78	3.38	10.37
IALD ^3^ by isolate Mle1	8.36	*Mle1-1*	OPE04b	1	131.45	12.2	15.47
		*Mle1-2*	ISSR15a	13	16.32	8.76	10.82
IALD ^3^ by isolate Mle2	5.88	*Mle2-1*	OPE04b	1	131.00	6.36	5.89
		*Mle2-2*	ISSR15d	2	55.32	5.37	4.95
		*Mle2-3*	OPC03a	12	8.78	9.40	8.86
IALD ^3^ by isolate Mle4	5.23	*Mle4-1*	OPA18k	1	22.00	14.09	10.15
		*Mle4-2*	ISSR17i	1	149.00	10.68	7.52
		*Mle4-3*	OPZ01c	2	15.32	19.50	14.57
		*Mle4-4*	OPE02b	5	24.79	17.05	12.53
		*Mle4-5*	OPZ19b	12	4.78	6.50	4.46
		*Mle4-6*	OPT12b	14	12.92	10.94	7.72

^1^ LG—Linkage Group; ^2^ %PVE—Phenotypic Variance Explained by QTL; ^3^ IALD—Infection Area of the Leaf Disc.

## References

[1] Karp A., Fenning T. (2014). Willows as a source of renewable fuels and diverse products. Challenges and Opportunities for the World's Forests in the 21st Century.

[2] Przyborowski J.A., Jędryczka M., Ciszewska-Marciniak J., Sulima P., Wojciechowicz K.M., Zenkteler E. (2012). Evaluation of the yield potential and physicochemical properties of the biomass of *Salix viminalis* × *Populus tremula* hybrids. Ind. Crops Prod..

[3] Stolarski M.J., Szczukowski S., Tworkowski J., Klasa A. (2013). Yield, energy parameters and chemical composition of short-rotation willow biomass. Ind. Crops Prod..

[4] Weih M., Singh B.P. (2013). 19 Willow. Biofuel Crops: Production, Physiology and Genetics.

[5] Karp A., Hanley S.J., Trybush S.O., Macalpine W., Pei M., Shield I. (2011). Genetic improvement of willow for bioenergy and biofuels. J. Integr. Plant Biol..

[6] Krzyżaniak M., Stolarski M.J., Waliszewska B., Szczukowski S., Tworkowski J., Załuski D., Śnieg M. (2014). Willow biomass as feedstock for an integrated multi-product biorefinery. Ind. Crops Prod..

[7] Hanley S.J., Karp A. (2013). Genetic strategies for dissecting complex traits in biomass willows (*Salix* spp.). Tree Physiol..

[8] Berlin S., Trybush S.O., Fogelqvist J., Gyllenstrand N., Hallingbäck H.R., Åhman I., Hanley S.J. (2014). Genetic diversity, population structure and phenotypic variation in European *Salix viminalis* L. (*Salicaceae*). Tree Genet. Genomes.

[9] Przyborowski J.A., Sulima P. (2010). The analysis of genetic diversity of *Salix viminalis* genotypes as a potential source of biomass by RAPD markers. Ind. Crops Prod..

[10] Trybush S.O., Jahodová Š., Čížková L., Karp A., Hanley S.J. (2012). High levels of genetic diversity in *Salix viminalis* of the Czech Republic as revealed by microsatellite markers. Bioenergy Res..

[11] Stolarski M.J., Szczukowski S., Tworkowski J., Wróblewska H., Krzyżaniak M. (2011). Short rotation willow coppice biomass as an industrial and energy feedstock. Ind. Crops Prod..

[12] Yang H., Li C., Lam H.M., Clements J., Yan G., Zhao S. (2015). Sequencing consolidates molecular markers with plant breeding practice. Theor. Appl. Genet..

[13] Berlin S., Lagercrantz U., von Arnold S., Öst T., Rönnberg-Wästljung A.C. (2010). High-density linkage mapping and evolution of paralogs and orthologs in *Salix* and *Populus*. BMC Genom..

[14] Ghelardini L., Berlin S., Weih M., Lagercrantz U., Gyllenstrand N., Rönnberg-Wästljung A.C. (2014). Genetic architecture of spring and autumn phenology in *Salix*. BMC Plant Biol..

[15] Hanley S., Mallott M., Karp A. (2006). Alignment of a *Salix* linkage map to the *Populus* genomic sequence reveals macrosynteny between willow and poplar genomes. Tree Genet. Genomes.

[16] Hanley S., Barker J., van Ooijen J., Aldam C., Harris S., Åhman I., Larsson S., Karp A. (2002). A genetic linkage map of willow (*Salix viminalis*) based on AFLP and microsatellite markers. Theor. Appl. Genet..

[17] Tsarouhas V., Gullberg U., Lagercrantz U. (2002). An AFLP and RFLP linkage map and quantitative trait locus (QTL) analysis of growth traits in *Salix*. Theor. Appl. Genet..

[18] Berlin S., Ghelardini L., Bonosi L., Weih M., Rönnberg-Wästljung A.C. (2014). QTL mapping of biomass and nitrogen economy traits in willows (*Salix* spp.) grown under contrasting water and nutrient conditions. Mol. Breed..

[19] Hallingbäck H.R., Fogelqvist J., Powers S.J., Turrion-Gomez J., Rossiter R., Amey J., Martin T., Weih M., Gyllenstrand N., Karp A. (2016). Association mapping in *Salix viminalis* L. (*Salicaceae*)-identification of candidate genes associated with growth and phenology. GCB Bioenergy.

[20] Salmon J., Ward S.P., Hanley S.J., Leyser O., Karp A. (2014). Functional screening of willow alleles in Arabidopsis combined with QTL mapping in willow (*Salix*) identifies SxMAX4 as a coppicing response gene. Plant Biotechnol. J..

[21] Weih M., Rönnberg-Wästljung A.C., Glynn C. (2006). Genetic basis of phenotypic correlations among growth traits in hybrid willow (*Salix dasyclados* × *S. viminalis*) grown under two water regimes. New Phytol..

[22] Samils B., Rönnberg-Wästljung A.C., Stenlid J. (2011). QTL mapping of resistance to leaf rust in *Salix*. Tree Genet. Genomes.

[23] Pucholt P., Rönnberg-Wästljung A.C., Berlin S. (2015). Single locus sex determination and female heterogamety in the basket willow (*Salix viminalis* L.). Heredity.

[24] Brereton N.J.B., Pitre F.E., Hanley S.J., Ray M.J., Karp A., Murphy R.J. (2010). QTL mapping of enzymatic saccharification in short rotation coppice willow and its independence from biomass yield. Bioenergy Res..

[25] Rönnberg-Wästljung A.C., Glynn C., Weih M. (2005). QTL analyses of drought tolerance and growth for a *Salix dasyclados × Salix viminalis* hybrid in contrasting water regimes. Theor. Appl. Genet..

[26] Hanley S.J., Pei M.H., Powers S.J., Ruiz C., Mallott M.D., Barker J.H., Karp A. (2011). Genetic mapping of rust resistance loci in biomass willow. Tree Genet. Genomes.

[27] Höglund S., Rönnberg-Wästljung A.C., Lagercrantz U., Larsson S. (2012). A rare major plant QTL determines non-responsiveness to a gall-forming insect in willow. Tree Genet. Genomes.

[28] Parker S.R., Royle D.J., Hunter T. (1993). Impact of *Melampsora* rust on yield of biomass willows. Proceedings of the 6th International Congress of Plant Pathology.

[29] Jędryczka M., Ciszewska-Marciniak J., Przyborowski J. (2008). The search for genetic sources of willow resistance to rust (*Melampsora epitea*). Phytopathol. Pol..

[30] Van Ooijen J. (2009). MapQTL 6. Software for the Mapping of Quantitative Trait Loci in Experimental Populations of Diploid Species.

[31] Rönnberg-Wästljung A.C., Samils B., Tsarouhas V., Gulberg U. (2008). Resistance to *Melampsora larici-epitea* leaf rust in *Salix*; analysis quantitative trait loci. J. Appl. Genet..

[32] Cochran W.G., Cox G.M. (1955). Experimental Designs.

[33] Shapiro S.S., Wilk M.B. (1965). An analysis of variance test for normality (complete samples). Biometrika.

[34] StatSoft, Inc 2014 STATISTICA (Data Analysis Software System), Version 12.5. http://www.statsoft.com.

[35] Feau N., Vialle A., Allaire M., Tanguay P., Hamelin R.C. (2009). Fungal pathogen (mis-) identifications: A case study with DNA barcodes on *Melampsora* rusts of aspen and white poplar. Mycol. Res..

[36] Sokal R.R., Rohlf F.J. (1995). Biometry: The Principles and Practice of Statistics in Biological Research.

[37] Sulima P., Przyborowski J.A., Załuski D. (2009). RAPD markers reveal genetic diversity in *Salix purpurea*. Crop Sci..

[38] Williams J.G.K., Kubelik A.R., Livak K.J., Rafalski J.A., Tingey S.V. (1990). DNA polymorphism amplified by arbitrary primers are useful as genetic markers. Nucleic Acids Res..

[39] Zietkiewicz E., Rafalski A., Labuda D. (1994). Genome fingerprinting by simple sequence repeat (SSR)-anchored polymerase chain reaction amplification. Genomics.

[40] McGregor C.E., Lambert C.A., Greyling M.M., Louw J.H., Warnich L. (2000). A comparative assessment of DNA fingerprinting techniques (RAPD, ISSR, AFLP and SSR) in tetraploid potato (*Solanum tuberosum* L.) germplasm. Euphytica.

[41] Broman K.W., Wu H., Sen S., Churchill G.A. (2003). R/QTL: QTL mapping in experimental crosses. Bioinformatics.

[42] Broman K.W., Sen S. (2009). A Guide to QTL Mapping with R/QTL.

[43] Xu S. (1996). Mapping quantitative trait loci using four-way crosses. Genet. Res..

[44] Lander E.S., Botstein D. (1989). Mapping Mendelian factors underlying quantitative traits using RFLP linkage maps. Genetics.

[45] Churchill G.A., Doerge R.W. (1994). Empirical threshold values for quantitative trait mapping. Genetics.

[46] Hoeschele I., van Raden P.M. (1993). Bayesian analysis of linkage between genetic markers and quantitative trait loci. I. Prior knowledge. Theor. Appl. Genet..

[47] Voorrips R.E. (2002). MapChart: Software for the graphical presentation of linkage maps and QTLs. J. Hered..

